# Oral microbiome in older adults with mild cognitive impairment

**DOI:** 10.1080/20002297.2023.2173544

**Published:** 2023-02-01

**Authors:** Dongxin Da, Qianhua Zhao, Hao Zhang, Wanqing Wu, Xiaoli Zeng, Xiaoniu Liang, Yiwei Jiang, Zhenxu Xiao, Jin Yu, Saineng Ding, Li Zheng, Ying Zhang, Xiaogang Xu, Ding Ding

**Affiliations:** a Department of Preventive Dentistry, Shanghai Stomatological Hospital& School of Stomatology, Fudan University, Shanghai, China; b Institute of Neurology, Huashan Hospital, Fudan University, Shanghai, China; c National Clinical Research Center for Aging and Medicine, Huashan Hospital, Fudan University, Shanghai, China; d National Center for Neurological Disorders, Huashan Hospital, Fudan University, Shanghai, China; e MOE Frontiers Center for Brain Science, Fudan University, Shanghai, China; f Shanghai Key Laboratory of Craniomaxillofacial Development and Diseases, Fudan University, Shanghai, China; g Institute of Antibiotics, Huashan Hospital, Fudan University, Shanghai, China

**Keywords:** Oral microbiome, mild cognitive impairment, older adults, 16S rDNA, bioinformatic analysis

## Abstract

The association between the oral microbiome and mild cognitive impairment (MCI) remains unclear. This study aimed to investigate such an association among Chinese older adults. Participants without dementia were recruited from the community. A battery of neuropsychological tests was administered to evaluate the cognitive function. The diagnosis of MCI was based on Peterson’s criteria. The non-stimulated saliva was collected to extract sequences of the oral microbiome. Forty-seven MCI and 47 cognitively normal participants were included. There was significant difference in alpha diversity and insignificant difference in beta diversity between the two groups of participants. Compared with the cognitively normal group, *Gemella haemolysans* and *Streptococcus gordonii* were two significantly decreased species while *Veillonella unclassified_Veillonella* and *Fusobacterium sp._HMT_203* were two significantly increased species in the MCI group. The richness of *Gemella haemolysans* presented the best discriminate value for MCI with the AUC (Area Under Curve) of 0.707, a cut-off value of 0.008 for relative abundance, the sensitivity of 63.8% and specificity of 70.2%. The dysbiosis of oral microbiome and relative abundance of *Gemella haemolysans* was significantly associated with MCI. Further studies were needed to develop new treatment strategies targeting the oral microbiome for cognitive impairment.

## Introduction

Alzheimer’s disease (AD) is a chronic progressive neurological disorder characterized by dysfunction of memory, thinking and behavior [1]. More than 55 million people had dementia in 2020. The number of people suffering from dementia is expected to double every 20 years, from 78 million in 2030 to 139 million in 2050, indicating a huge burden on society and families [2].

Oral microbes maintain homeostasis normally, but oral microbial dysbiosis would lead to oral diseases, such as periodontitis and dental caries, and even systemic chronic inflammation [3]. An interventional study also suggested a significant reduction in systemic inflammation after periodontal treatment [4]. Oral microbes are believed to cause neurological diseases through the direct effect of infection or indirect effect of inflammation [5,6]. Systemic inflammation prevented the nervous system from clearing abnormal cells and proteins, leading to the accumulation of amyloid plaques and neurofibrillary tangles, and ultimate cognitive decline [7]. Kamer et al. found the elevation of serum levels of TNF-α and antibodies against periodontal pathogens in AD patients compared to controls [8]. Díaz-Zúñigaet al. found that LPS of *Actinobacillus actinomycetemcomitans* promoted neurological inflammation by activating microglia, leading to an increase in inflammatory cytokines associated with Aβ42 accumulatio [9]. Poole et al. reported the presence of periodontal bacterial in AD patients and suggested a potential link between oral microflora dysbiosis and AD [10].

Mild cognitive impairment (MCI) is a reversible stage between cognitively normal and dementia [1]. Several studies found that 30% − 50% of individuals initially diagnosed with MCI reverted to ‘normal’ cognition at subsequent follow-up [11,12]. Han et al. found MCI patients with impairments/in multiple cognitive domains were less likely to revert to normal than those with impairments in a single domain [11]. Despite the possibility of reverting to normal, 5%–10% of MCI patients convert to dementia, which is much higher than that in the general population (1%–2% incidence rate) [13]. Early detection and prevention is necessary to reduce the burden on patients and families brought by MCI and dementia. Since oral microbiome might be associated with MCI, the study of the oral microbes of MCI and normal groups may improve further understanding of the pathogenesis of MCI and promote early detection and prevention of MCI. Moreover, most previous studies were hospital-based case–control studies, and few were community-based studies with more representative samples.

This study aims to explore the composition and structure of oral microbiome in individuals with MCI comparing with cognitively normal ones enrolled from the community, and to evaluate the discriminate value of oral microbes for individuals with MCI.

## Materials and methods

### Study population

A total of 157 older adults aged ≥60 living in the community in downtown Shanghai were recruited. After propensity score matching, we included 47 MCI and 47 cognitively normal participants in the study. Participants were excluded if they (1) suffered from cancer, diabetes with complications, rheumatoid arthritis, severe schizophrenia or developmental delay or intellectual disability; (2) had severe vision, hearing, or speaking problems, wearing oral removable dentures and were unable to cooperate with the neuropsychological and oral health evaluation; (3) were diagnosed with dementia.

The Medical Ethics Committee of Huashan Hospital affiliated to Fudan University approved the study (approval number: HIRB2009–195). Written informed consents were obtained from all participants and/or their legally acceptable representatives.

### Social-demographic data

Face-to-face questionnaire surveys were conducted by nurses to collect the social-demographics and lifestyle characteristics of the participants. Data including age, sex, education, height, weight, alcohol drinking and medical histories were collected. Body Mass Index (BMI) was calculated with height and weight. Medical histories including hypertension and diabetes were confirmed with medical records.

### Neuropsychological assessments and diagnosis

A battery of neuropsychological tests was adopted to assess the cognitive function of the participants. The cognitive functions included global cognition, memory, attention, executive function, spatial construction and language. The neuropsychological test battery contained 1) Mini-Mental State Examination; 2) Conflicting Instructions Task (Go/No Go Task); 3) Stick Test; 4) Modified Common Objects Sorting Test; 5) Auditory Verbal Learning Test; 6) Modified Fuld Object Memory Evaluation; 7) Trail-making Tests A and B; and 8) RMB (Renminbi) Test. Psychometrists conducted the tests based on the participants’ education levels in Chinese within 90 minutes. The details of the tests above had been published elsewhere [14,15].

The diagnosis of MCI was based on the Peterson criteria [16]: 1) complaints of cognitive impairment, or manifestations of cognitive impairment detected by informed persons, nurses, and physicians; 2) objective evidence of cognitive decline in at least one cognitive domain based on performance 1.5 SD below the mean using the norms obtained in the previous population-based study [14]; 3) basic ability to care for oneself in daily life; and 4) failure to meet diagnostic criteria for dementia of DSM-IV.

### Saliva collection and oral examination

According to the Human Microbiome Project (HMP) [17], participants were not allowed to eat, smoke or drink alcohol 30 minutes before sampling after getting up in the morning. The dentist opened the sterile Eppendorf tube and collected 2 ml non-stimulated saliva. Then, the tube was marked and stored in the −80°C refrigerator immediately.

One dentist completed the oral examinations of all participants according to the methods in the WHO guidelines [18]. Number of missing teeth excluding the third molar and DMFT was calculated. DMFT was the sum of decayed, missing and filled teeth.

### DNA extraction, PCR amplification and 16S rDNA sequencing

Total genomic DNA was extracted using DNA Extraction Kit following the manufacturer’s instructions. Quality and quantity of DNA was verified by NanoDrop and agarose gel. Extracted DNA was diluted to a concentration of 1 ng/μl and stored at-20°C until further processing. The diluted DNA was used as template for PCR amplification of bacterial 16S rDNA genes with the barcoded primers and Takara Ex Taq (Takara). V3-V4 variable regions of 16S rDNA genes were amplified with universal primers 343F (5′-TACGGRAGGCAGCAG-3′) and 798 R (5′-AGGGTATCTAATCCT-3′).

Amplicon quality was visualized using gel electrophoresis, purified with AMPure XP beads (Agencourt), and amplified for another round of PCR. After purified with the AMPure XP beads again, the final amplicon was quantified using Qubit dsDNA assay kit. Equal amounts of purified amplicon were pooled and sequencing of the 16S amplicon was performed by OE Biotech Co., Ltd. on an Illumina MiSeq with two paired-end read cycles of 300 bases each.

### Bioinformatic sequence analysis

Raw sequencing data were in FASTQ format. Paired-end reads were then preprocessed using Trimmomatic software [19] to detect and cut off ambiguous bases (N). It also cut off low-quality sequences with average quality score below 20 using sliding window trimming approach.

After trimming, paired-end reads were assembled using FLASH software [20]. Parameters of assembly were: 10bp of minimal overlapping, 200bp of maximum overlapping and 20% of maximum mismatch rate.

Sequences were performed further denoising as follows: reads with ambiguous, homologous sequences or below 200bp were abandoned. Reads with 75% of bases above Q20 were retained. Then, reads with chimera were detected and removed. These two steps were achieved using QIIME software (version 1.8.0) [21].

Clean reads were subjected to primer sequences removal and clustering to generate operational taxonomic units (OTUs) using Vsearch software with 97% similarity cutoff [22].

The representative read of each OTU was selected using QIIME package. All representative reads were annotated and blasted against HOMD database Version 15.2 (16s rDNA).

The calculation of absolute and relative abundance, alpha and beta diversity, principal component analysis (PCA) and principal coordinate analysis (PCoA) was done in R software (version 4.1.2, R Foundation for Statistical Computing) using the ‘vegan’ and ‘picante’ packages. Diversity parameters were calculated from OTUs that were rarefied to an equal number per sample to reduce the effect of different sequencing depth using ‘vegan’ package. Unweighted pair-group Method with arithmetic means (UPGMA) was done by ‘phangorn’ package and online tools were used for LEfSe (Linear discriminant analysis coupled with effect size measurements) [23]. Analysis concerning ROC was completed by ‘pROC’ package.

### Statistical analysis

For the current data, relative enrichness of *Gemella haemolysans* in MCI and cognitively normal group were 0.007 and 0.011. With the sample size 47 in each group, the power of test (1 − β) was 0.903 with α = 0.05using ‘pwr’ package in R software (version 4.1.2, R Foundation for Statistical Computing), hence the sample size was enough in our study.

Quantitative variables were expressed as mean and standard deviation, while categorical variables were expressed as frequency (%). Samples satisfying normal distribution and homogeneity of variance were compared using the t-test, and others were compared using the Kruskal–Wallis test. We used the chi-square test to compare the frequencies. *P* < 0.05 was considered statistically significant. Statistical analysis was performed using SAS 9.4 (SAS Institute Inc., Cary, NC, USA).

## Results

### Characteristics of study participants

Forty-seven participants with MCI and 47 cognitively normal participants were recruited. The sociodemographic data and medical histories are showed in Table 1. Age, gender, years of education, BMI, history of hypertension and diabetes, number of missing teeth and DMFT were not significantly different between MCI and cognitively normal groups (*P* > 0.05).

**Table 1.** Demographic, lifestyle and medical history between participants with MCI and normal cognition.

	ALL (*N* = 94)	MCI (*N* = 47)	Cognitively normal (*N* = 47)	P value*
Male, n(%)	46(48.94)	23(50.00)	23(50.00)	1.00
Age,years,mean±sd	73.58 ± 5.52	74.05 ± 6.09	73.12 ± 4.90	0.45
Education,years,mean±sd	12.15 ± 3.40	11.64 ± 4.05	12.66 ± 2.54	0.12
Body mass index,mean±sd	24.36 ± 3.22	24.39 ± 3.04	24.33 ± 3.43	0.93
Alcohol drinking, n (%)	10(10.64)	4(8.51)	6(12.77)	0.74
Hypertension, n(%)	50(53.19)	20(42.55)	30(63.83)	0.06
Diabetes mellitus, n(%)	15(15.96)	9(19.15)	6(12.77)	0.57
DMFT, mean±sd	14.67 ± 9.74	15.87 ± 10.20	13.44 ± 9.18	0.24
Missing teeth, mean±sd	10.97 ± 9.50	12.45 ± 10.40	9.46 ± 8.32	0.13

*Comparison between participants with MCI and normal cognition.

### Sample sequencing

In this study, non-stimulated saliva samples were collected from 94 participants and each sample contained between 10,961 and 57,165 clean tags after sequencing and quality control. The number of valid tags obtained from clean tags after removing chimera ranged from 9478 to 52,093. Sequences were divided into many OTUs (Operational Taxonomic Units) according to their similarity. After removing low-confidence OTUs, a total of 1904 OTUs were finally obtained, with an average of 231.09 OTUs for each sample. The number of OTUs in each sample was between 75 and 331.

### Analysis of bacterial diversity

Table 2 showed the alpha diversity indices for the samples. The sequencing depth (Observed species index and Coverage index) and richness (Chao1 index) between the two groups were significantly different (*P* < 0.05). There was no significant difference in evenness (Shannon and Simpson index) (*P* > 0.05). Although the coverage index of the two groups was statistically significant, they were both close to 100% and the mean difference was merely 1%. The Chao1 index of cognitively normal group was higher than that of MCI group, indicating that the number of taxa present in the ecological community of normal cognition group is richer, which was consistent with the result suggested by observed Species index. There was no significant difference between Shannon index and Simpson index, suggesting the similarity of the distribution and abundance of taxa between the two groups.

**Table 2.** Alpha diversity indices of both groups.

	ALL	MCI	Cognitively normal	P value*
Observed species, mean±sd	308.83 ± 63.64	292.45 ± 61.15	325.21 ± 62.45	0.01
Coverage,mean±sd	0.99 ± 0.00	0.99 ± 0.00	0.99 ± 0.00	<0.01
Chao1,mean±sd	459.40 ± 110.13	426.56 ± 97.55	492.23 ± 113.14	<0.01
Shannon, mean±sd	5.52 ± 0.49	5.50 ± 0.48	5.54 ± 0.50	0.70
Simpson,mean±sd	0.95 ± 0.02	0.95 ± 0.02	0.95 ± 0.02	0.70

* Comparison between participants with MCI and normal cognition.

### Abundance and distribution of oral microbiome

**Figure 1.**
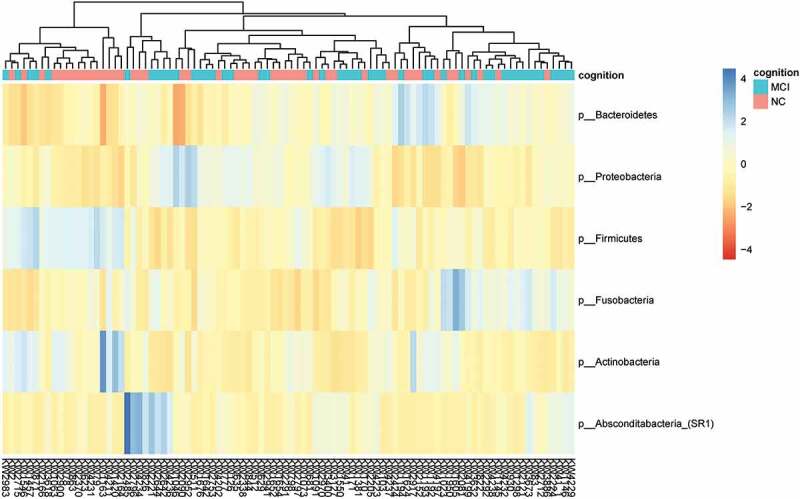
Taxonomic distribution of the top 10 relative abundant phyla in the participants with MCI and normal cognition.

**Figure 2.**
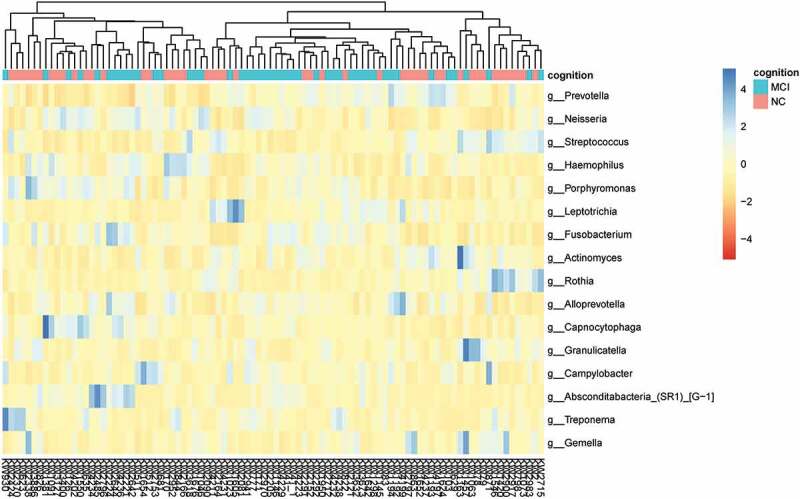
Taxonomic distribution of the top 10 relative abundant genera in the participants with MCI and normal cognition.

There was a total of 13 phyla, 33 classes, 57 orders, 103 families, 201 genera and 1904 species in the samples. Figures 1 and 2 showed the top 6 dominant phyla and top 16 dominant genera of classification, respectively. While we used relative abundance >1% as dominant, eight dominant phyla were *Bacteroidetes* (33.96%), *Proteobacteria* (22.38%), *Firmicutes* (21.03%), *Fusobacteria* (10.15%), *Actinobacteria* (9.66%), *Absconditabacteria_(SR1)* (1.26%), with the sum of the relative abundance reaching 98.45%. The 16 dominant genera were *Prevotella* (20.93%), *Neisseria* (12.10%), *Streptococcus* (10.13%), *Haemophilus* (6.96%), *Porphyromonas* (6.18%), *Leptotrichia* (5.38%), *Fusobacterium* (4.64%), *Actinomyces* (4.32%), *Rothia* (4.10%), *Alloprevotella* (2.85%), *Capnocytophaga* (2.69%), *Granulicatella* (2.27%), *Campylobacter* (1.63%), *Absconditabacteria_(SR1)_[G-1]* (1.26%), *Treponema* (1.12%), and *Gemella* (1.12%), reaching 87.70% in total relative abundance. The above 16 genera were also dominant in each of the two groups.

### Composition and structure of oral microbiome

**Figure 3.**
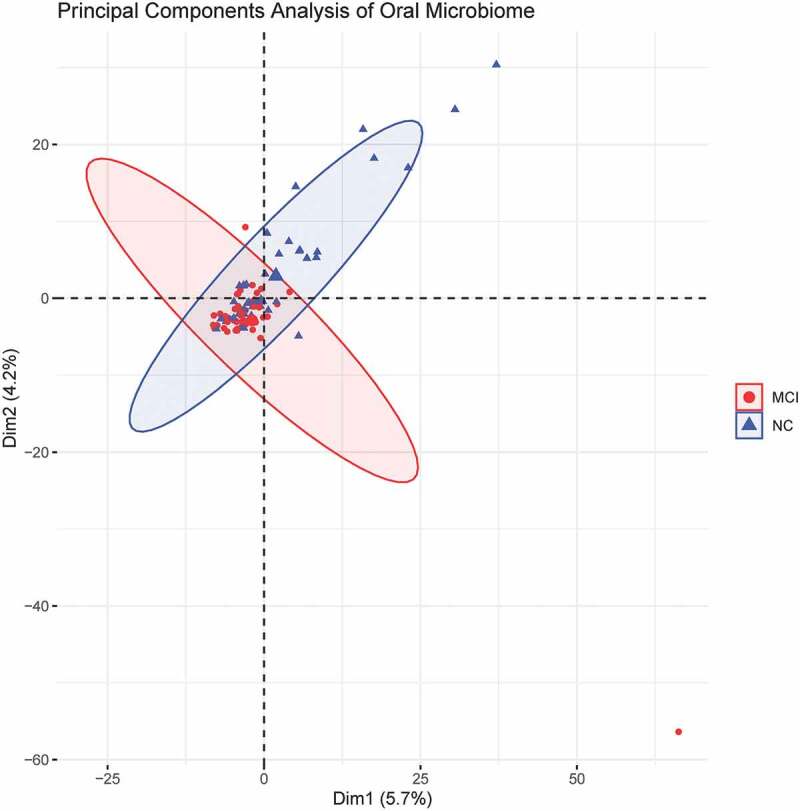
Principal component analysis showed different beta diversities indicating different microbiome structure between the MCI and cognitively normal groups.

**Figure 4.**
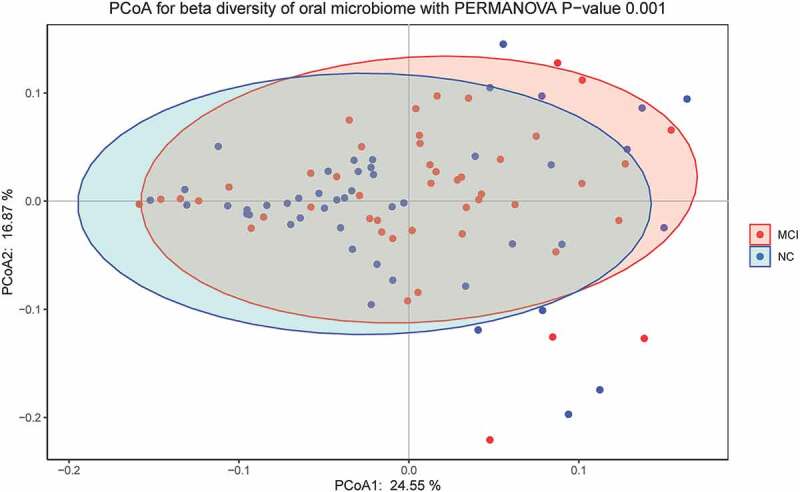
Principal coordinates analysis showed different beta diversities indicating different microbiome structure between the MCI and cognitively normal groups.

**Figure 5.**
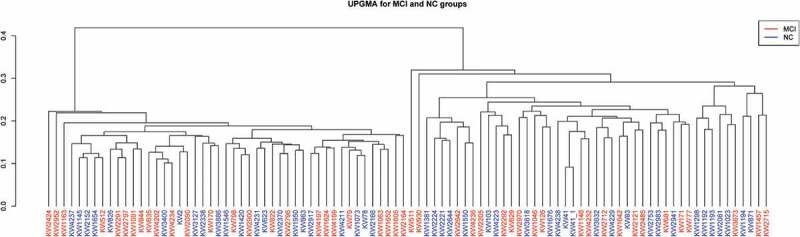
Upgma(unweighted pair-group method with arithmetic means) showed different beta diversities indicating unsimilar microbiome structure between the MCI and cognitively normal groups.

In this study, PCoA based on Bray-Curtis distance was used to analyze the beta diversity of MCI and normal cognitive groups. The results of PCoA, PCA and UPGMA clustering analysis showed that the structure of ecological communities of the two groups were different (Figures 3, 4 and 5). The results of permutational multivariate analysis of variance (PERMANOVA) indicated the significant difference between bacterial community structures of the two groups (*P* = 0.001).

### Species as potential indicators

**Figure 6.**
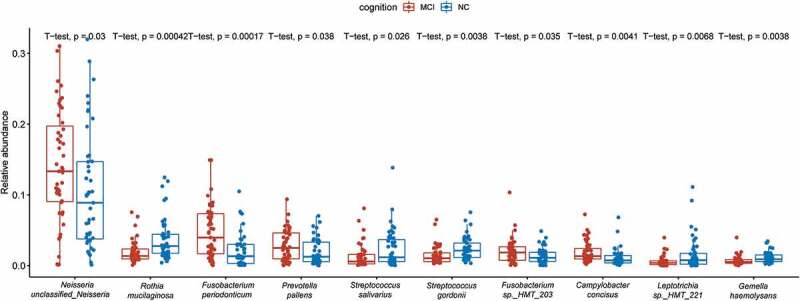
Boxplot of the top 10 abundant differentiated genera in the MCI and cognitively normal groups.

**Figure 7.**
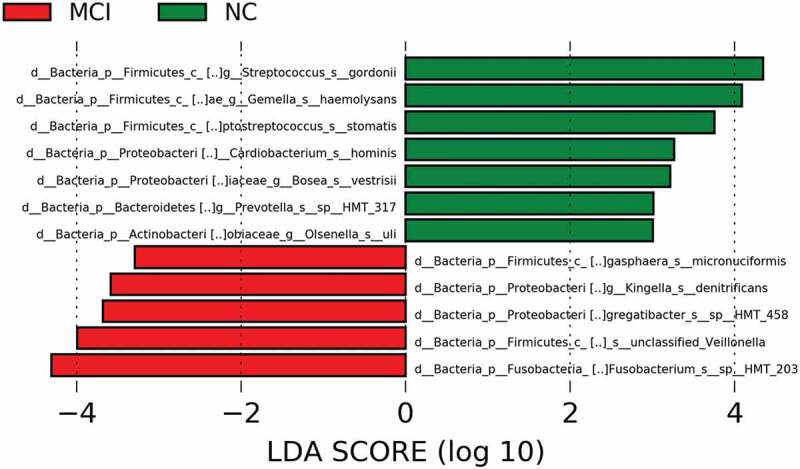
Legends showed composition of different genera of microbiome between the participants with MCI and normal cognition using LEfSe analysis.

In this study, we selected 10 most differently abundant species by t test (Figure 6). The 10 species with the most significant differences were *Neisseria unclassified_Neisseria, Rothia mucilaginosa, Fusobacterium periodonticum, Prevotella pallens, Streptococcus salivarius, Streptococcus gordonii, Fusobacterium sp._HMT_203, Campylobacter concisus, Leptotrichia sp._HMT_221, and Gemella haemolysans*. LEfSe analysis was also used in this study to more accurately analyze the differences between the two groups. LEfSe analysis revealed the different composition of different species in microbial communities between the two groups (Figure 7). The phylogenetic tree showed that the significantly enriched species in cognitively normal group were *Streptococcus gordonii, Gemella haemolysans, Peptostreptococcus stomatis, Cardiobacterium hominis, Bosea vestrisii, Prevotella sp._HMT_317 and Olsenella uli*, while those in MCI group were *Megasphaera micronuciformis, Kingella denitrificans, Aggregatibacter sp._HMT_458, Neisseria unclassified_Neisseria* and *Fusobacterium sp._HMT_203*.

**Figure 8.**
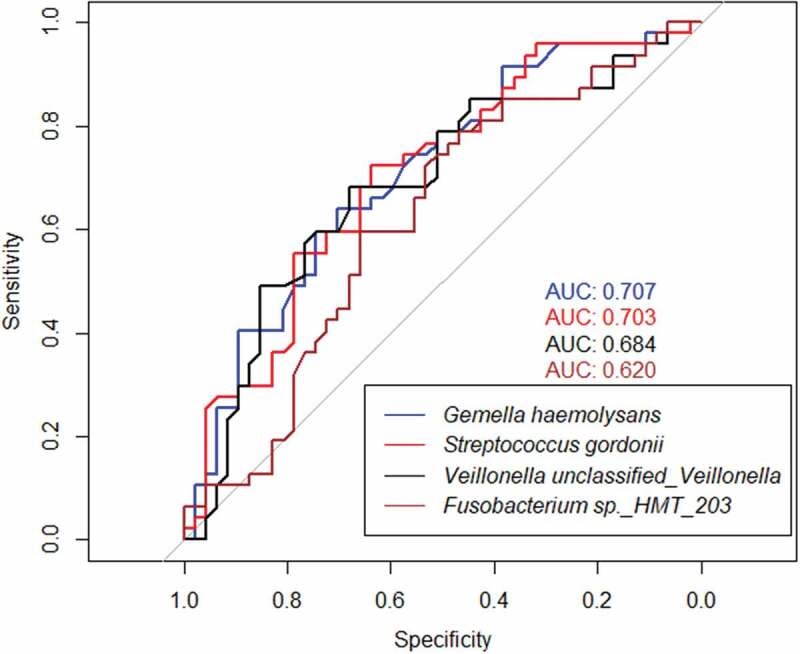
Rocs of *Gemella haemolysans* (AUC:0.707), *Streptococcus gordonii* (AUC:0.703), *Veillonella unclassified_veillonella* (AUC:0.684) and *Fusobacterium sp._hmt_203* (AUC:0.620) for the discrimination of MCI.

The above significantly different microbes could be used as potential indicators for each group (LDA>3, *P* < 0.05). The ROC curve was made by using each of the above taxa as discriminative indicators. The AUC of *Gemella haemolysans* was the largest (0.707), with a cut-off value of 0.008 for relative abundance, the sensitivity of 63.8% and specificity of 70.2% (Figure 8). The AUC of *Gemella haemolysans and Streptococcus gordonii* was significantly higher than that of *the rest species*(*P* < 0.05), but there was no significant difference between the AUC of the two above (*P* > 0.05).

## Discussion

In the current study, we found six most dominant phyla in the samples and the top five were *Bacteroidetes, Proteobacteria, Firmicutes, Fusobacteria, Actinobacteria*, which was consistent with the five core phyla founded in previous studies [24–26]. The most dominant 6 phyla and 16 genera including the above 5 were also most abundant in both MCI and normal cognitive groups, suggesting MCI did not alter the major composition of the oral microbiome. Our results were also consistent with a previous cross-sectional study [25].

Microbiome diversity could be described by alpha and beta diversities. The significant difference in observed species and Chao1 index suggested that altered number of taxa might be associated with MCI. However, no significant difference in Shannon and Simpson index indicated that the distribution of taxa was not correlated with MCI. Beta diversity, as the root of microbiome community structure research, was often used to compare differences between ecosystems and to reflect the heterogeneity of biological species. Beta diversity analysis could be conducted by many methods, including PCA, PCoA and UPGMA. PCA and PCoA were based on the similarity coefficient matrix and distance matrix of samples to downscale multidimensional data to compare difference between groups, respectively. UPGMA was based on the distance matrix using hierarchical cluster analysis to construct a tree structure for visualization. The results of PCoA, PCA and UPGMA showed significant difference of beta diversity between the two groups. The results were in partial consistence with a smaller sampled study which found no significant difference in alpha and beta diversity between MCI and normal cognitive groups [25].

Despite the overall similarity of alpha and beta diversity, we characterized some differently abundant taxa in both groups. In the results of LEfSe, *Gemella haemolysans* and *Streptococcus gordonii* were two species significantly enriched in cognitively normal individuals with the maximal AUC. *Gemella haemolysans* was a Gram-indeterminate bacteria found in the human oral, intestinal and respiratory tracts. Tomohiro Miyoshi et al. have found the protein components in the culture supernatant of *Gemella haemolysans* directly suppressed the growth of *P. gingivalis* [27]. *P. gingivalis* was the major pathogens of periodontitis. Several studies had proved the association between these periodontal pathogens and cognitive dysfunction [28–30]. In the brains of AD patients, Dominy et al. found *P. gingivalis* and its virulence factor, gingipains, whose levels were correlated with amyloid plaques [28]. Bacteria of the genus *Streptococcus* are acquired at birth and the first to colonize the oral cavity. *Streptococcus gordonii* could produce much alkali that neutralize the acid-producing action of *Streptococcus mutans* [31]. *Streptococcus gordonii* also possesses two paralogs of AgI/II, SspA and SspB, mediating its adhesion to human and bacterial receptors [32].

Several previous studies have demonstrated the association between oral periodontal pathogens and cognitive decline. Periodontitis is a chronic inflammation caused by dental plaque in periodontal tissues, and the ‘red complex’ plays an important role in the formation of dental plaque biofilm [33]. The ‘red complex’ was periodontal pathogens including *Porphyromonas gingivalis, Tannerella forsythia* and *Treponema denticola* [34]. However, inconsistent with previous studies, three species belonging to ‘red complex’ were not found to be significantly different in both groups. There were two reasons as the explanation. First, the structure of salivary microbiome might be different from that in peripheral blood, cerebrospinal fluid, and brain. Second, most of the above studies only focused on one or two species of bacteria. Confounding factors including the microbial interaction might not be controlled.

This study had several limitations. Firstly, the sensitivity and specificity of Fusobacterium were not satisfying due to the relatively small sample size. Longitudinal studies with larger sample sizes are needed to demonstrate its potential as an indicator. Secondly, our results suggested a possible association via a case–control design. Further studies are needed to investigate the causal relationship between oral microbiome and MCI. Thirdly, existing software and databases of microbiome cannot completely identify all sequences, therefore unrecognized or misrecognized sequences may still exist. However, we have tried to minimize these problems by using the latest versions of software and databases. Fourthly, we collected oral microbiomes from saliva because saliva had easy access to digestive tracts and was the main source of microbes in dental plaque. The structures of oral microbiomes are highly correlated with sites, which might be the cause of difference between saliva and dental plaque. The conclusion of this study cannot be directly generalized to dental plaque. However, the salivary microbiome was readily available and similar to dental plaque in ecological distribution and structure, and the significant association between cognitive impairment and microbiome from dental plaque has been found in several previous studies [25,35,36].

## Conclusion

In this case–control study, we demonstrated the structure and difference of oral microbiome between MCI and normal cognitive groups. The alpha and beta diversity of salivary microbiome in individuals with MCI was significantly different comparing with normal cognitive ones in some aspects. We found a group of taxa correlated with MCI, among which *Gemella haemolysans* might have the closest association. It suggested that the prevention and treatment of cognitive dysfunction should also include keeping the oral microecological balance, preventing periodontitis and promoting oral health. Further studies are needed to develop new treatment strategies targeting the oral microbiome for cognitive impairment.

## Data Availability

The authors can share their relevant raw data supporting their findings. If any scientist wishes to use them for non-commercial purposes, without breaching participant confidentiality, he or she can contact the authors directly, and they will share their raw data freely with him or her.
